# Immunopathological Mechanisms of Bird-Related Hypersensitivity Pneumonitis

**DOI:** 10.3390/ijms24032884

**Published:** 2023-02-02

**Authors:** Silvia Sánchez-Díez, María Jesús Cruz, Miquel de Homdedeu, Iñigo Ojanguren, Christian Romero-Mesones, Irene Sansano, Xavier Muñoz

**Affiliations:** 1 Pulmonology Service, Department of Medicine, Vall d’Hebron University Hospital, Autonomous University of Barcelona, 08035 Barcelona, Spain; 2 CIBER of Respiratory Diseases (CIBERES), 28029 Madrid, Spain; 3 Pathological Anatomy Service, Vall d’Hebron University Hospital, Autonomous University of Barcelona, 08035 Barcelona, Spain; 4 Department of Cell Biology and Physiology and Immunology, Autonomous University of Barcelona, 08193 Barcelona, Spain

**Keywords:** hypersensitivity pneumonitis, murine model, pigeon, cytometry

## Abstract

Bird-related hypersensitivity pneumonitis (BRHP) is an interstitial lung disease induced by avian proteins. The immunopathological pathways involved in the disease are still unknown. This study assesses the cellular immune response and the cytokine pattern in a mouse model of BRHP. On days −3 and −1, mice were intraperitoneally sensitized with commercial pigeon serum (PS) or saline. Intranasal instillations with PS or saline were carried out on three consecutive days/week over either 3 weeks (Group 1) or 12 weeks (Group 2). Leukocyte and cytokine patterns in lung tissue and pulmonary inflammation in bronchoalveolar lavage (BAL) were analysed. Both groups presented increases in resident monocytes, interstitial macrophages and type 2 dendritic cells (DCs), but also reductions in inflammatory monocytes, alveolar macrophages and tolerogenic DCs compared with their control groups. Group 1 had increased levels of eosinophils and T cells with reductions in neutrophils and B cells, while Group 2 showed high levels of B cells. Both groups exhibited increases in Th1 and Th2 cytokines. Group 2 also showed increased levels of IL-23, a Th17 cytokine. Increased levels of neutrophils, eosinophils and lymphocytes were observed in BAL samples of both groups compared with controls. In the first stages of BRHP, there is a mixed Th1/Th2 immune response, while during the progression of the disease, although there is a Th1 response, the cytokine levels seem to indicate a switch towards a Th2/Th17 mixed response.

## 1. Introduction

Hypersensitivity pneumonitis (HP) is an interstitial lung disease characterized by an inflammation of the lung parenchyma, alveoli and bronchioles in susceptible individuals due to the inhalation of a wide variety of organic and inorganic compounds, usually protein antigens of microorganisms, fungi or animals [1,2]. There are two different categories of this disease: acute/inflammatory and chronic/fibrotic. The acute form appears with intermittent but high-level antigen exposure, is often reversible with antigen avoidance and is related to cellular inflammation. The chronic type appears with continuous but low-dose antigen exposure, is reversible only in some cases and is characterized by fibrotic areas inside the lungs [3]. Symptoms common to both forms include dyspnoea, cough and midinspiratory squeaks. Bird-related hypersensitivity pneumonitis (BRHP) is the most common type of HP and it occurs after inhalation exposure to avian antigens [4]. 

The immunopathological pathways of BRHP are still unknown, and this fact makes the diagnosis of the disease more complex. In this context, identifying an early-stage immune response to HP will help clinicians to improve the diagnostic accuracy and prognosis of patients with the disease. Thus, by providing insight about its pathophysiological mechanisms, we can avoid the progression of the disease to a chronic and irreversible form with pulmonary fibrosis, a condition which results in significant loss of lung function with the development of respiratory failure that leads to death if a lung transplant is not carried out. Even though the pathophysiological mechanisms involved in HP are poorly understood, there is some evidence of the contribution of both humoral and cellular immune responses. Antigen presentation by innate immune cells to B lymphocytes induces the production of immunoglobulins (Igs) [2,5]. These Igs can bind to antigenic proteins forming immunocomplexes, which can activate the classical pathway of the complement and induce tissue injury [6]. Antigen-presenting cells also interact with T cells, leading to the secretion of different cytokines such as IL-12 and IFN-γ and stimulating the polarization of lymphocytes to Th1 cells [7]. The secretion of IL-17 by lymphocytes and neutrophils induces a Th17 immune response, which also contributes to chronic inflammation [8]. Some studies have demonstrated the presence of T regulatory cells with an impaired function, which also play a role in the exaggerated immune response [9,10]. In later stages of HP, a relative switch of Th1 cells to Th2 promotes the maintenance of inflammation and the development of fibrosis [11,12]. The decreasing apoptosis of lymphocytes in these final stages also contributes to T cell persistence, activation and accumulation in lung tissue while the apoptosis of alveolar epithelial cells and granulocytes stimulates chemokine production by dendritic cells (DCs), leading to an increased recruitment of immune cells in the lungs [13]. The maintenance of these inflammatory patterns, together with the abnormal activation of fibroblasts, contributes to pulmonary fibrosis [14].

In a previous study of a mouse model of HP, analysing bronchoalveolar lavage (BAL) samples, our group identified the role of neutrophils in the development of the disease and an evolution towards a Th2 immune response in later stages of the disease [15]. In an attempt to shed light on the immunomodulatory mechanisms of HP, the present study assesses the cellular immune response and the cytokine pattern involved in the disease via the analysis of lung tissue.

## 2. Results

### 2.1. Flow Cytometry 

**Figure 1:**
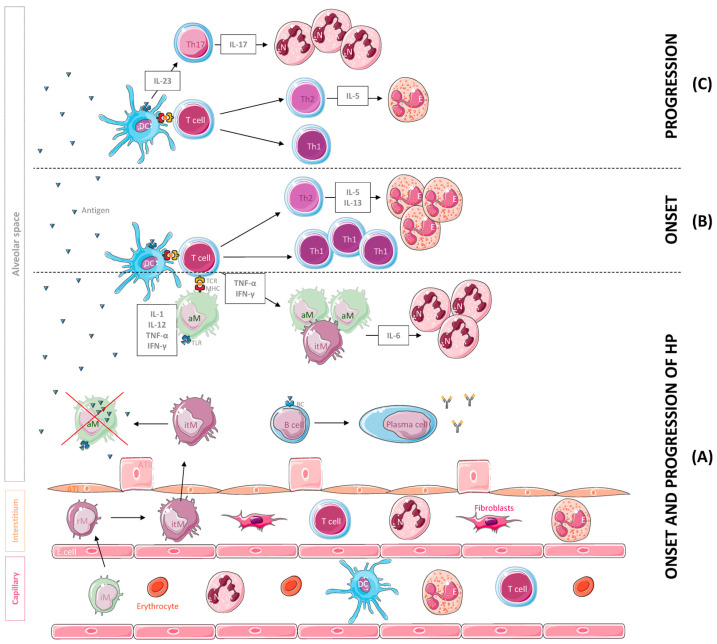
Leukocyte population and cytokines mainly involved in the onset and the progression of hypersensitivity pneumonitis (HP). (**A**) Following inhalation, the antigen is phagocytosed and degraded by dendritic cells (DCs) and macrophages and coupled to MHC molecules. The antigen is then recognized by T cells that secrete cytokines such as IFN-γ and TNF-α, inducing the accumulation, activation and aggregation of IL-6-producing macrophages, a potent chemoattractant for neutrophil (N) recruitment and activation. A B cell response against the antigens also occurs, leading to the production of specific antibodies. For their part, alveolar macrophages (aMs) may undergo subsequent apoptosis after the phagocytosis of high levels of the antigen and inflammatory monocytes (iMs), which are recruited to the lung tissue from the vasculature and differentiate into resident monocytes (rMs) and interstitial macrophages (itMs) afterwards. Subsequently, itMs proliferate and migrate into the alveolar space to restore this depleted population. (**B**) In the onset of the disease, T cells differentiate into Th1 and Th2 effectors, and Th2 cells release cytokines such as IL-5 and IL-13 that promote eosinophil recruitment and activation. (**C**) With progression of the disease, Th2 cells decrease IL-5 secretion, resulting in a reduction in eosinophils in the site of inflammation. In addition, DCs start secreting IL-23, a cytokine that promotes Th17 cell differentiation, IL-17 secretion and neutrophil aggregation. Abbreviations: ATI, alveolar type I cell; ATII, alveolar type II cell; E. cell, endothelial cell.

**Figure 2:**
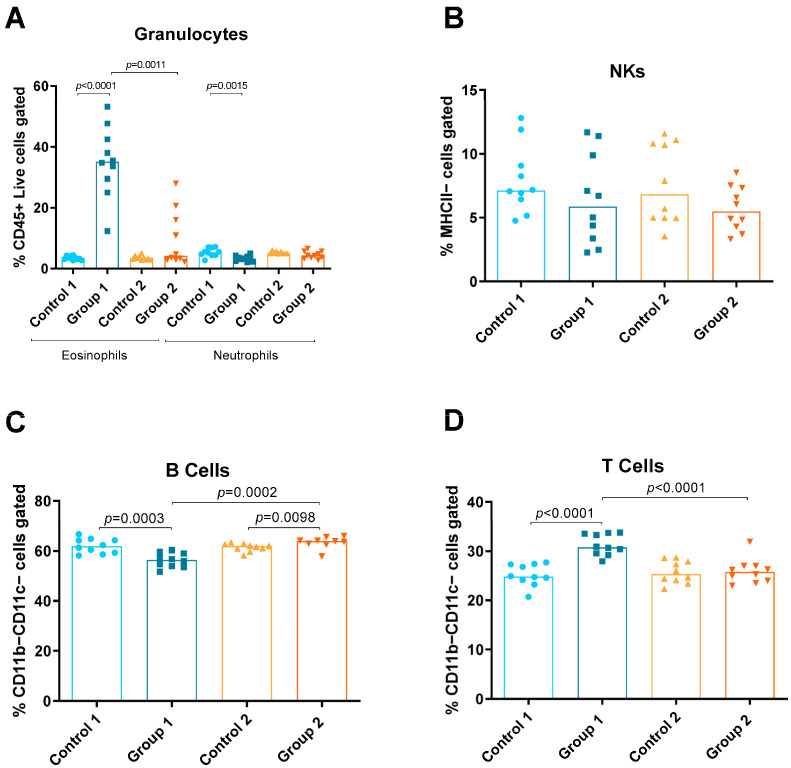
Granulocyte, natural killer (NK) cell and lymphocyte levels in lung homogenates from flow cytometry analysis. Experimental groups are the same as in Figure in Section 4.3. In each group, data are shown as individual and median values of eosinophils and neutrophils (**A**), NK cells (**B**), B cells (**C**) and T cells (**D**).

**Figure 3:**
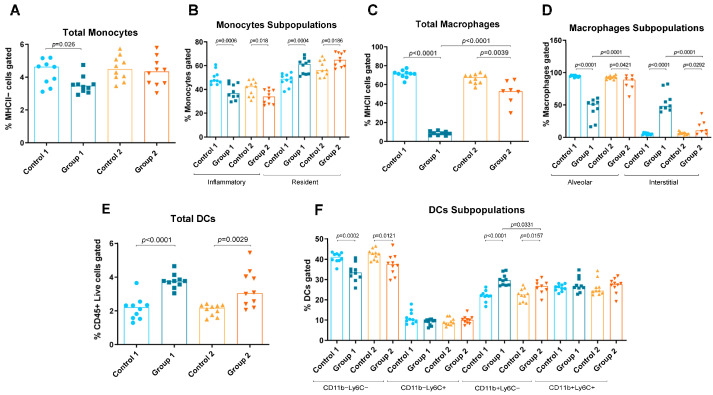
Monocyte, macrophage and dendritic cell (DC) levels in lung homogenates from flow cytometry analysis. Experimental groups are the same as in Figure in Section 4.3. In each group, data are shown as individual and median values of total monocytes (**A**), inflammatory and resident monocytes (**B**), total macrophages (**C**), alveolar and interstitial macrophages (**D**), total DCs (**E**), and CD11b−Ly6C−, CD11b−Ly6C+, CD11b+Ly6C− and CD11b+Ly6C+ DCs (**F**).

Using the panel described, the relative amounts of the following different immune cells in lung tissue were discriminated and quantified: eosinophils, neutrophils, natural killers (NKs), B and T cells, monocytes (total, inflammatory and resident), macrophages (total, alveolar and interstitial) and DCs (total, CD11b−Ly6C− CD11b−Ly6C+, CD11b+Ly6C− and CD11b+Ly6C+) (Figure 1, Figure 2 and Figure 3). Group 1 exhibited increases in eosinophils and T cells compared with the control group (*p* < 0.0001 and *p* < 0.0001, respectively) (Figure 2A,D). A decrease in neutrophils and B cells (*p* = 0.0015, *p* = 0.0003, respectively) was also observed in Group 1 compared with its control group (Figure 2A,C, respectively). In Group 2, a statistically significant increase was observed in B cells (*p* = 0.0098) compared with the control (Figure 2C). When comparing Groups 1 and 2, reductions in eosinophils and T cells (*p* = 0.0011 and *p* < 0.0001, respectively) (Figure 2A,D) and an increase in B cells (*p* = 0.0002) were observed (Figure 2C). 

Group 1 also exhibited increases in resident monocytes, interstitial macrophages, T cells, total DCs and CD11b+Ly6C− DCs (*p* = 0.0004, *p* < 0.0001, *p* < 0.0001, *p* < 0.0001 and *p* < 0.0001, respectively) compared with the AC control group (Figure 2D and Figure 3B,D–F). Reductions in total and inflammatory monocytes, total and alveolar macrophages and CD11b−Ly6C−DCs (*p* = 0.026, *p* = 0.0006, *p* < 0.0001, *p* < 0.0001 and *p* = 0.0002, respectively) were also observed in Group 1 compared with its control group (Figure 3A–D,F). In Group 2, statistically significant increases were observed in resident monocytes, interstitial macrophages and total DCs and CD11b+Ly6C− DCs (*p* = 0.0186, *p* = 0.0292, *p* = 0.0029 and *p* = 0.0157, respectively) compared with the control group (Figure 3B,D–F). Group 2 also exhibited reductions in inflammatory monocytes, total and alveolar macrophages and CD11b−Ly6C− DCs (*p* = 0.018, *p* = 0.039, *p* = 0.0421 and *p* = 0.0121, respectively) compared with its control group (Figure 3B–D,F). When comparing Groups 1 and 2, reductions in interstitial macrophages and CD11b+Ly6C− DCs (*p* < 0.0001 and *p* = 0.0331, respectively) (Figure 3D and Figure 3F, respectively) and increases in total and alveolar macrophages (*p* < 0.0001 and *p* < 0.0001, respectively) were observed (Figure 3C and Figure 3D, respectively).

### 2.2. Cytokine Level Analysis

**Figure 4:**
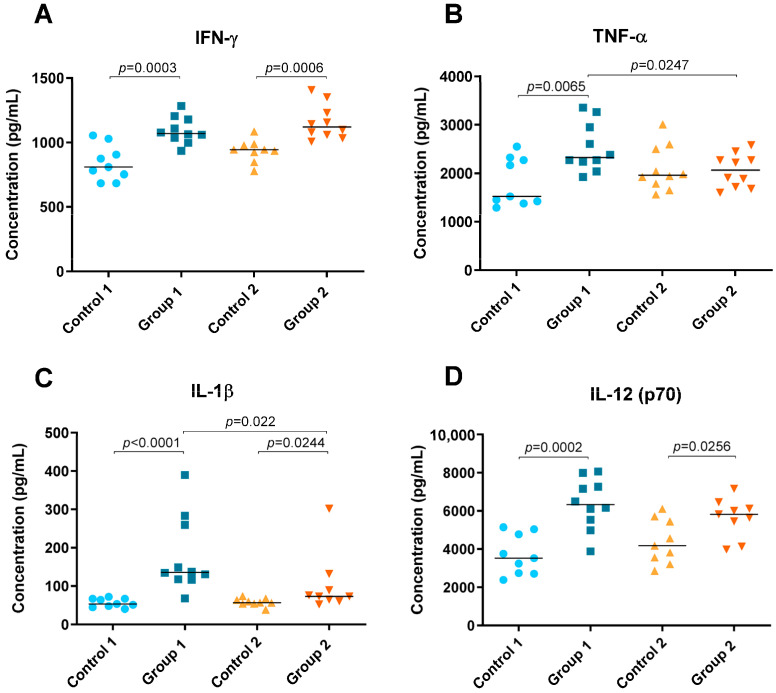
Th1 cytokine concentration (pg/mL) in lung tissue homogenate. In each group, data are shown as individual and median values of IFN-γ (**A**), TNF-α (**B**), IL-1β (**C**) and IL-12 (p70) (**D**).

**Figure 5:**
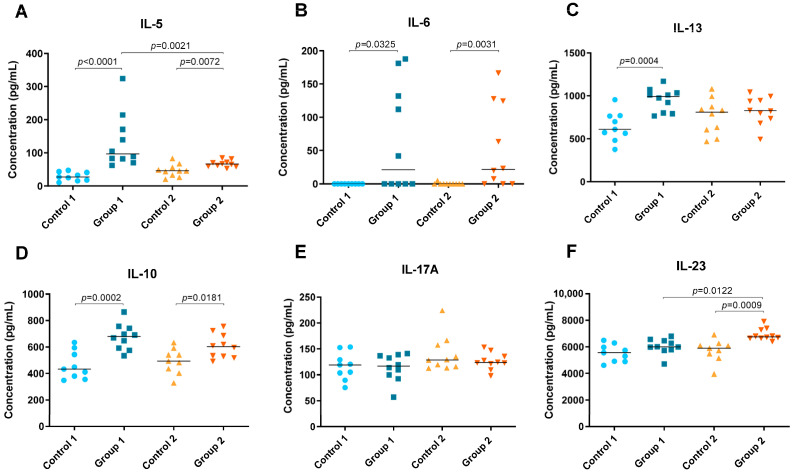
Th2 and Th17 cytokine concentration (pg/mL) in lung tissue homogenate. In each group, data are shown as individual and median values of IL-5 (**A**), IL-6 (**B**), IL-13 (**C**), IL-10 (**D**), IL-17A (**E**) and IL-23 (**F**).

Levels of cytokines IFN-γ, TNF-α, IL-1β, IL-5, IL-6, IL-10, IL-12 (p70), IL-13, IL-17A and IL-23 were detected in lung tissue homogenate and are shown in Figure 4 and Figure 5. In both groups of the disease, significant increases were observed in Th1 cytokines such as IFN-γ (*p* = 0.0003 and *p* = 0.0006, respectively), IL-1β (*p* < 0.0001 and *p* = 0.0244, respectively) and IL-12 (p70) (*p* = 0.0002 and *p* = 0.0256, respectively) compared with their respective control groups (Figure 4A,C,D). However, the concentration of IL-1β was higher in Group 1 than in Group 2 (*p* = 0.022) (Figure 4C). Significant differences were also observed for TNF-α between Group 1, its control group and Group 2, being higher in the group exposed to pigeon serum for less time (*p* = 0.0065 and *p* = 0.0247, respectively) (Figure 4B).

In both groups, Groups 1 and 2, significant increases were observed in Th2 cytokines such as IL-5 (*p* < 0.0001 and *p* = 0.0072, respectively) and IL-6 (*p* = 0.0325 and *p* = 0.0031, respectively) but also in IL-10 (*p* = 0.0002 and *p* = 0.0181, respectively) (Figure 5A,B,D). However, the concentration of IL-5 was higher in Group 1 than in Group 2 (*p* = 0.0021) (Figure 5A). Significant differences were also observed between Group 1 and its control group for IL-13, which was higher in the group exposed to pigeon serum (*p* = 0.0004) (Figure 5C). The concentration of IL-23 was higher in Group 2 than in the respective control and Group 1 (*p* = 0.0009 and *p* = 0.0122, respectively) (Figure 5F). No significant differences were observed for IL-17A between groups (Figure 5E).

### 2.3. Total and Differential Cell Counts in BAL

**Figure 6:**
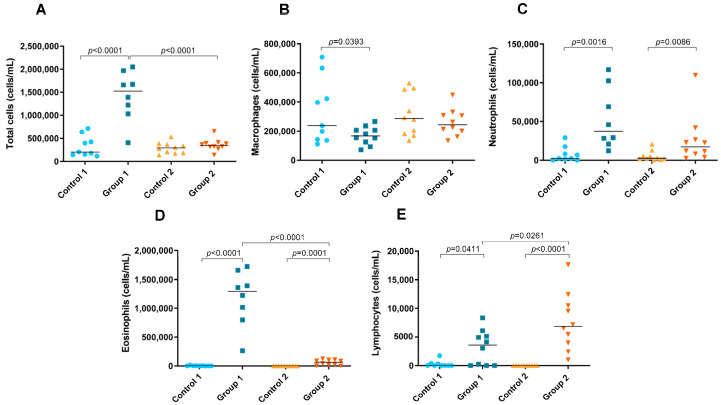
Total and differential cell counts in bronchoalveolar lavage. In each group, data are shown as individual and median values of total cells (**A**), macrophages (**B**), neutrophils (**C**), eosinophils (**D**) and lymphocytes (**E**).

A significant increase in the number of total cells in BAL fluid was observed in Group 1 compared with its control group (*p* < 0.0001) (Figure 6A). In addition, the total cell count was significantly higher in the group exposed for less time to pigeon serum than in Group 2 (*p* < 0.0001) (Figure 6A). In Group 1, a significant decrease in the number of macrophages was observed compared with its control group (*p* = 0.0393) (Figure 6B). In both groups of the disease, increases in the neutrophil (*p* = 0.0016 and *p* = 0.0086, respectively), eosinophil (*p* < 0.0001 and *p*= 0.0001, respectively) and lymphocyte counts (*p* = 0.0411 and *p* < 0.0001, respectively) were observed compared with the control groups (Figure 6C–E, respectively). However, the number of eosinophils was significantly lower in Group 2 than in Group 1 (*p* < 0.0001) (Figure 6D), while the lymphocyte count was higher (*p* = 0.0261) (Figure 6E).

### 2.4. Histopathological Analysis

**Figure 7:**
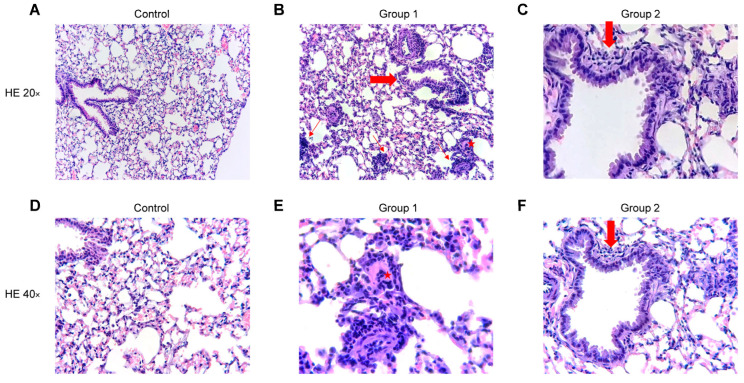
Representative images of haematoxylin and eosin (HE) stained histological lung sections. Experimental groups in this figure are represented with sections at 20× and 40× magnification from control group (**A**,**D**), Group 1 (**B**,**E**) and Group 2 (**C**,**F**). (**A**,**D**) Normal lung parenchyma. (**B**) Lung tissue with cellular bronchiolitis (big arrow), patchy interstitial pneumonitis (thin arrows) and giant cell (star). (**E**) Higher magnification of the giant cell (star). (**C**,**F**) Lung tissue with mild cellular bronchiolitis (big arrow).

The blinded histopathological examination of lung tissue sections revealed mild to severe (grade 1–3) bronchiolitis and peribronchiolar inflammation in Group 1 (Figure 7B,E) and mild to moderate (grade 1–2) inflammation in Group 2 (Figure 7C,F). In both groups, a mild to moderate (grade 1–2) interstitial inflammation was observed with the presence of perivascular lymphocyte cuffing, giant cells and arteriolar muscularization. The degree of inflammation fell in Group 2 and no peribronchiolar or interstitial fibrosis was found. No inflammatory infiltrate was observed in the control group (Figure 7A,D). No significant differences were observed between Groups 1 and 2 after rating the pathological findings in a semi-quantitative way.

## 3. Discussion

The present study assesses the effect of repeated exposure to pigeon serum in the onset and progression of BRHP in a mouse model, by characterizing the immune responses in the lung. Our results suggest that in the onset of the disease there is a mixed Th1/Th2 immune response characterized by eosinophil infiltration, Th2-related DC recruitment and a Th1/Th2 cytokine pattern. On the other hand, after 12 weeks of repeated exposure a switch towards Th2/Th17 mixed response takes place with notable recruitment of lymphocytes, macrophages and Th2-related DCs, and the release of Th2 and Th17 cytokines.

Using the flow cytometry panel previously described [16] and after BAL analysis, we were able to compare the leukocyte pattern of the onset and the progression of BRHP in lung tissue. Inhalation of pigeon serum antigen for three weeks produced a type 2 response with increased levels of eosinophils, resident monocytes and CD11b+Ly6C− DCs. Moreover, high concentrations of interstitial macrophages and T cells were observed. The main differences between the onset and the progression of BRHP were that eosinophil recruitment was much more evident in the first stages of the disease, with a reduction in B cells after short-term exposure to pigeon serum while the lymphocyte count was higher with disease progression. The increased eosinophilia in Group 1 may be related to the short time span (24 h) between the last antigen exposure and the lung sample acquisition [17,18]. Along the same lines, in a study in 30 patients with HP, Drent M et al. [18] also discussed whether activated IL-5-producing CD4+ T cells were responsible for the eosinophil recruitment in the airways after recent exposure to the causative antigen. This suggestion is also supported by our study, in which a significant increase in IL-5 in lung tissue was observed in Group 1. In fact, a previous study by our group [15] supports this hypothesis because increases in IL-5 were observed in BAL samples after three weeks of exposure. In addition, another study carried out by our group in patients with HP also demonstrated a pattern of eosinophil inflammation in induced sputum samples after a specific inhalation challenge to avian antigens [19]. In Group 2, eosinophil inflammation was practically absent, coinciding with reductions in IL-5, the major hematopoietic cytokine regulating eosinophil proliferation and survival [20], and in IL-13, which has been associated with eosinophil activation, chemoattraction and survival in vitro [21,22].

The increased number of lymphocytes observed after long-term exposure to the antigen is in accordance with previous reports [23]. In particular, marked lymphocytosis has already been identified as a typical finding in BAL samples of patients with HP, especially in the chronic form [4,23,24]. The high levels of lymphocytes observed, especially after 12 weeks of repeated exposure to pigeon serum, may be associated with the increasing levels of IL-23. This interleukin has been described as a critical cytokine in the development of chronic inflammation via the activation of IL-17-producing T cells and neutrophil recruitment [25,26,27]. In this connection, the current study also identified neutrophil inflammation in BAL samples of mice in Group 2, and several previous studies have demonstrated the involvement of neutrophils in the progression of the disease and the development of fibrosis [8,28]. In particular, Hasan et al. [8] concluded that, in experimental HP, neutrophils and monocytes/macrophages are the predominant sources of IL-17, a cytokine involved in pulmonary fibrosis. The lower levels of B cells observed in Group 1 may be due to the differentiation of B cells into plasma cells that secrete antibodies against the inhaled antigen [29]. In fact, immunoglobulins have been implicated in the early phases of the disease forming antigen–antibody complexes and are detected in serum samples of patients with HP [30,31,32].

The present study provides further evidence that pigeon serum exposure reduces alveolar macrophages and inflammatory monocytes while increasing resident monocytes and interstitial macrophages in both the acute and progressive models. These findings suggest that alveolar macrophages may undergo subsequent apoptosis and that inflammatory monocytes may be recruited to the lung tissue from the vasculature and differentiate into resident monocytes and interstitial macrophages afterwards. Subsequently, interstitial macrophages may proliferate and migrate into the alveolar space to restore this depleted population [33,34].

DCs are considered to be the link between innate and adaptive immune responses, inducing either tolerance or immunity to foreign antigens. The effect of pigeon serum on CD11b−Ly6C− DCs is also evident in this BRHP model. These type 1 classical dendritic cells have been reported to induce tolerance to inhaled antigens and to phagocyte apoptotic epithelial cells, and to cross-present them via MHC class I to CD8+ T cells in the lung-draining bronchial lymph node [35,36]. Both groups of the disease had significantly reduced levels of CD11b−Ly6C− DCs, suggesting that pigeon serum induces lung epithelial damage and decreases airway tolerance, leading to an exaggerated immune response. Another subset of dendritic cells that seems to play an important role in the BRHP model is that of CD11b+Ly6C− DCs. These type 2 classical dendritic cells (cDC2) are specialized in MHC class-II-mediated antigen presentation and have been previously reported to have the ability to trigger a Th2-cell-mediated immune response to inhaled allergens [37]. However, cDC2 can also promote Th1 and Th17 cell responses in specific contexts via the secretion of proinflammatory cytokines such as IL-12 and IL-23 [38,39]. In the present study, an increase in IL-12 was observed in Groups 1 and 2 while levels of IL-23 were only increased in mice with disease progression. These results support the hypothesis that with BRHP progression a Th17 immune response is being activated by CD11b+Ly6C− DCs, while in the onset of the disease these cells promote a type 1 response with the released cytokines.

The changes observed in the immunological pattern during the onset and the progression of HP could have a clinical benefit for patients suffering from this disease. In this sense, the results obtained could provide insights into personalized precision medicine for patients with HP according to the stage of the disease. Therefore, clinicians could adapt the treatment of these patients based on their immunological profile, for instance, by using monoclonal antibodies against Th1- or Th2-related cytokines.

The main limitation of this study is the use of a nontraditional model for chronic HP. In this sense, the present study attempts to broaden our knowledge of the immune pathways involved in the onset and progression of HP. However, further studies are needed to develop understanding of the chronic form of the disease in order to assess the immunological mechanisms that evolve into lung fibrosis.

## 4. Materials and Methods

### 4.1. Antigen Solution

Commercial pigeon serum (Rockland Immunochemicals Inc., Pottstown, PA, USA) with a protein concentration of 15.5 mg/mL, determined previously by the bicinchoninic acid (BCA) method (Pierce Chemical Co., Rockford, IL, USA), was used to induce the disease. This antigenic extract demonstrated an adequate reproducibility and availability in previous studies [40]. Specifically, aliquots with a concentration of 200 µg protein/mL were prepared as the antigen solution.

### 4.2. Animals

Male C57BL/6JOlaHsd mice (~23 g, 6 weeks old) were obtained from Envigo (Horst, The Netherlands). Mice were housed in individually ventilated cages in a conventional animal house with 12 h dark/light cycles, and received slightly acidified water and pelleted food (Teklad diet, 2014; Envigo, Indianapolis, IN, USA) ad libitum. One group of mice was used to analyse the leukocyte pattern in lung tissue using flow cytometry and to determine cytokine levels in lung tissue homogenate. The other set of mice was used to assess total and differential cell counts using BAL and histopathological features in lung tissue. All experimental procedures were approved by the local Ethical Committee for Animal Experiments of the Vall d’Hebron Research Institute (ID: N7Q5YTNDJ; 7 May 2021).

### 4.3. Experimental Design

**Figure 8:**
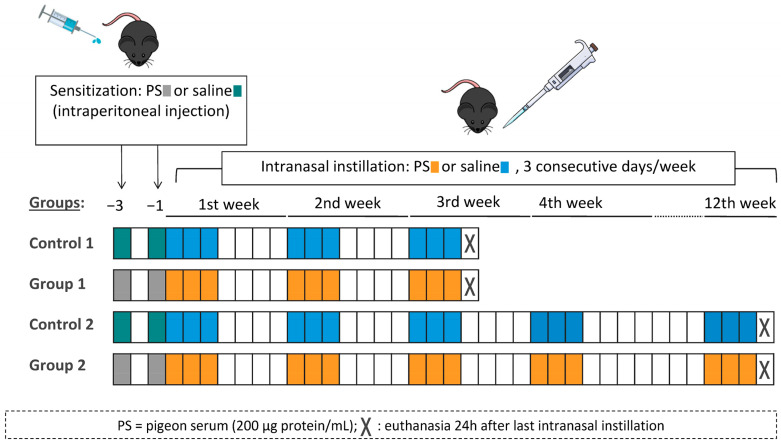
Flow chart of the experimental design and the four experimental groups. Mice were sensitized by intraperitoneal injection with 100 µL of pigeon serum (PS) or saline on days −3 and −1 and then received intranasal instillations of 40 µL of PS or saline on three consecutive days/week for 3 weeks (Group 1) or 12 weeks (Group 2). Mice were sacrificed 24 h after the last intranasal instillation.

The experimental design was similar to the one previously described by our group [15]. In each round of the model, mice were randomly divided into four groups (six mice per group). On days −3 and −1, mice were sensitized with an intraperitoneal injection of 100 µL of commercial pigeon serum (200 μg protein/mL) or vehicle (saline, 0.9% NaCl). On day 0, under light anaesthesia with isoflurane (Forane, Abbott Laboratories, Madrid, Spain), mice received intranasal instillations of 40 μL of pigeon serum (200 μg protein/mL) or vehicle (saline, 0.9% NaCl). Intranasal instillations were carried out on three consecutive days per week for either 3 weeks (Control 1 and Group 1) or 12 weeks (Control 2 and Group 2). Mice were euthanized 24 h after the last intranasal instillation. The experimental groups were Control 1 (sensitized and challenged with saline), Group 1 (sensitized and challenged with pigeon serum), Control 2 (sensitized and challenged with saline) and Group 2 (sensitized and challenged with pigeon serum). A diagram of the experimental design is shown in Figure 8.

### 4.4. Flow Cytometry

The first set of mice were treated, 24 h after the last intranasal instillation, with 50 μL of 1000 U/mL s.c. heparin and deeply anaesthetized with isoflurane (Forane, Abbott Laboratories, Madrid, Spain). A cytometry protocol previously described by our group was applied [16]. Briefly, each mouse was perfused with cold PBS (phosphate-buffered saline) through the heart’s right atrium with a 21-gauge metal needle. Lungs were carefully removed and preserved on ice. The left lobule and one of the right lobules were minced and digested with digestion solution containing collagenase A (Roche Diagnostics S.L., Basel, Switzerland) and DNaseI (Roche Diagnostics S.L., Basel, Switzerland). Digested lobules were filtered through a 70 µm cell strainer (Corning Incorporated, New York, NY, USA), treated with ACK lysing buffer (Invitrogen, Carlsbad, CA, USA) and washed twice. After counting cells with an automated cell counter (LUNA-II, Logos Biosystems, Villeneuve d’Ascq, France), 10^6^ cells were stained with Fixable Viability Stain 510 (FVS510, BD Biosciences, San Jose, CA, USA), washed twice and incubated with Brilliant Stain Buffer and purified rat anti-mouse CD16/CD32 (BD Biosciences, San Jose, CA, USA). After this incubation, APC-R700 rat anti-CD11b, BV786 hamster anti-mouse CD11c, APC-Cy7 rat anti-mouse CD45, BV605 rat anti-mouse I-A/I-E, PE rat anti-mouse CD24, BV650 rat anti-mouse Ly-6G (BD Biosciences, San Jose, CA, USA), Brilliant Violet 421 anti-mouse CD64 (BioLegend, San Diego, CA, USA) and PerCP-Cyanine5.5 Ly-6C (eBioscience, San Diego, CA, USA) monoclonal antibodies were added. Unstained controls were analysed with each stained sample. Data were acquired using an LSR Fortessa cell analyser (BD Biosciences, San Jose, CA, USA) and analysed using FlowJo software (version X 10.0.7r2, TreeStar, Ashland, OR, USA). Gating strategy used for the identification of immune cells in lung tissue is shown in Supplementary Figure S1.

### 4.5. Lung Tissue Homogenate for Cytokine Level Analysis

Lung tissue homogenate was obtained by homogenizing 40 mg of mouse lung tissue with 200 µL of lysis buffer (Procartaplex Cell Lysis Buffer, Invitrogen, MA, USA) containing 15% protease inhibitors (Complete™ Mini Protease Inhibitor Cocktail, Merck, Darmstadt, Germany). After centrifugation at 16,000× *g* for 10 min at 4 °C, the supernatant was collected and its protein concentration was determined using the BCA method (Pierce Chemical Co., Rockford, USA). Supernatants were then stored at −80 °C until cytokine analysis.

Levels of interferon gamma (IFN-γ), tumour necrosis factor alpha (TNF-α) and interleukin-1β (IL-1β), IL-5, IL-6, IL-10, IL-12 (p70), IL-13, IL-17A and IL-23 were measured in the supernatant of lung tissue homogenate with mouse cytokine magnetic bead panels (Bio-Plex Pro Mouse Cytokine Assay, Bio-Rad Laboratories S.A., Madrid, Spain) according to the manufacturer’s instructions. Lower limits of detection were 1.84, 5.8, 10.36, 3.57, 0.74, 2.95, 1.62, 47.2, 2.65 and 3.4 pg/mL for IFN-γ, TNF-α, IL-1β, IL-5, IL-6, IL-10, IL-12 (p70), IL-13, IL-17A and IL-23, respectively.

### 4.6. Bronchoalveolar Lavage for Total and Differential Cell Counts

In the other set of mice, bronchoalveolar lavage was performed as previously described by our group [16]. Briefly, lungs were washed three times with 0.7 mL of sterile saline. Total cells were counted using an automated cell counter (LUNA-II, Logos Biosystems, France) and the BAL fluid was centrifuged at 1000× *g* for 10 min. The supernatant was stored at −80 °C until further analyses and the pellet was resuspended in PBS for differential cell counts. A volume of 100 µL of resuspended cells adjusted to 0.75 × 10^6^ cells/mL were spun at 450 rpm for 6 min (Cytospin™4 cytocentrifuge, Thermo Fisher Scientific, Waltham, MA, USA) onto microscope slides. Slides were air-dried, fixed in methanol for 10 min and stained with May–Grünwald (Química Clínica Aplicada S.A, Tarragona, Spain) for 5 min and 30% Giemsa (Merck, Darmstadt, Germany) for 15 min. Counts of macrophages, neutrophils, eosinophils, basophils and lymphocytes were carried out in a total of 400 cells from each sample in a blinded manner.

### 4.7. Histopathological Analysis

After BAL sample collection, lungs were removed. The left lungs were frozen in liquid nitrogen and stored at −80 °C for future studies while the right lobules were immediately stored in 4% formaldehyde for histopathological analysis. Right lobules were then fixed in 10% buffered formalin for 24 h, sectioned and processed by dehydration in ascending series of alcohol and embedding in paraffin wax. Next, 3 μm thick sections were obtained from the paraffin blocks and stained with haematoxylin and eosin (HE). The histological examination was performed by a pathologist in a blinded manner using an optical microscope (Leica DM 2000 LED, Leica Microsystems, Wetzlar, Germany). Lung injury was scored according to the following variables: centrilobular inflammation, interstitial inflammation, bronchiolitis, peribronchiolar fibrosis, presence of giant cells and interstitial fibrosis. These features were graded using 4-point scales: 0 = regular tissue, 1 = mild changes, 2 = moderate changes, 3 = significant changes. Presence or absence of giant cells and arteriolar muscularization was also recorded. Pictures were taken from representative findings at 20× and 40× magnification. An example photo of each score found from representative findings is provided in Supplementary Figure S2.

### 4.8. Statistical Analysis

Data are shown as mean and standard deviation or as individual data and group medians, as appropriate. Parametric and nonparametric statistics were performed according to data distribution, which was evaluated using Shapiro–Wilk test. Multiple comparisons between groups were performed using one-way ANOVA followed by Tukey’s post hoc test or the Kruskal–Wallis test followed by Dunn’s multiple comparisons, according to the data distribution. Analyses were conducted using GraphPad Prism 6 for Windows (version 6.01, GraphPad Software Inc., San Diego, CA, USA) and IBM SPSS Statistics (version 26, IBM Corporation, Armonk, New York, NY, USA). Differences with a *p*-value < 0.05 (two-tailed) were considered to be significant.

## 5. Conclusions

In conclusion, the present study suggests that in the first stages of BRHP there is a mixed Th1/Th2 immune response, and that with the progression of the disease, although there is a Th1 response, the levels of cytokines seem to indicate a switch towards a Th2/Th17 mixed response.

## Data Availability

Data supporting reported results will be made available on request.

## Supplementary Materials

The supporting information can be downloaded at: https://www.mdpi.com/article/10.3390/ijms24032884/s1.
