# Supplementary material for: Immunopathological Mechanisms of Bird-Related Hypersensitivity Pneumonitis
Source: Int J Mol Sci. 2023 Feb 2;24(3):2884. doi: 10.3390/ijms24032884 (PMC9917634; doi:10.3390/ijms24032884)
Supplement: Supplementary file 1 [file ijms-24-02884-s001.zip › ijms-2131601-supplementary.pdf]

International Journal of
Molecular Sciences

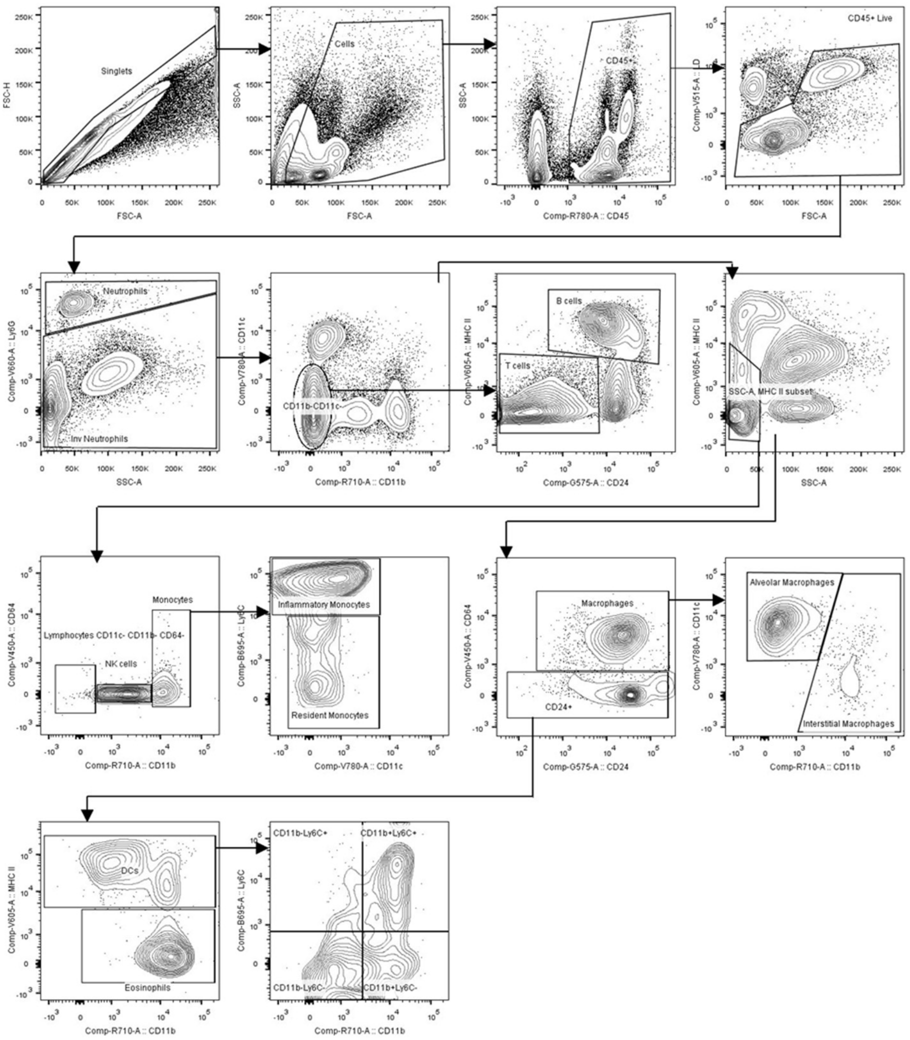

Supplementary Figure S1. Gating strategy used at flow cytometry analysis for the identification of immune cells in lung tissue.

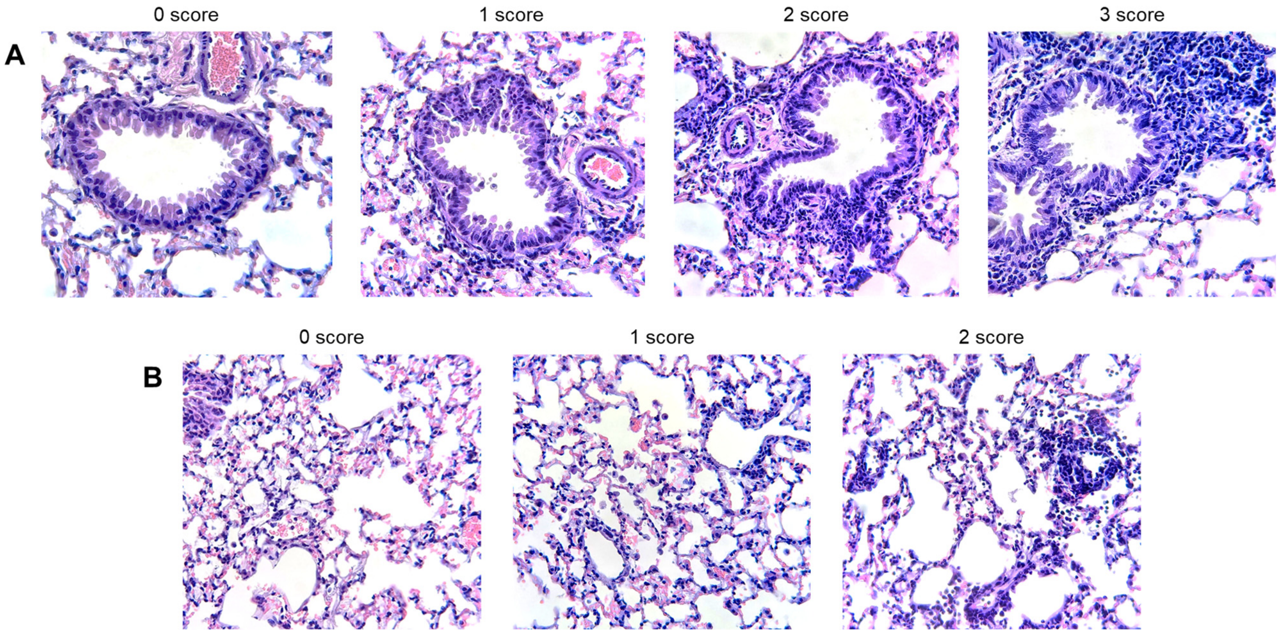

Supplementary Figure S2. Representative images of each score found (0: regular, 1: mild, 2: moderate and 3: significant changes) of cellular bronchiolitis (A) and interstitial inflammation (B) at 40x magnification in haematoxylin and eosin (HE) stained histological lung sections.
